# Imaging aortic wall inflammation^☆^^✰✰^

**DOI:** 10.1016/j.tcm.2018.12.003

**Published:** 2019-11

**Authors:** Maaz B.J. Syed, Alexander J. Fletcher, Marc R. Dweck, Rachael Forsythe, David E. Newby

**Affiliations:** Department of Cardiovascular Sciences, Queens Medical Research Institute, University of Edinburgh, 49 Little France Crescent, Edinburgh EH16 4TJ, United Kingdom

## Abstract

Inflammation affects the aortic wall through complex pathways that alter its biomechanical structure and cellular composition. Inflammatory processes that predominantly affect the intima cause occlusive disease whereas medial inflammation and degeneration cause aneurysm formation.

Aortic inflammatory pathways share common metabolic features that can be localized by smart contrast agents and radiolabelled positron emission tomography (PET) tracers. ^18^F-Fluorodeoxyglucose (^18^F-FDG) is a non-specific marker of metabolism and has been widely used to study aortic inflammation in various diseased aortic states. Although useful in detecting disease, ^18^F-FDG has yet to demonstrate a reliable link between vessel wall disease and clinical progression.

^18^F-Sodium fluoride (^18^F-NaF) is a promising biological tracer that detects microcalcification related to active disease and cellular necrosis within the vessel wall. ^18^F-NaF shows a high affinity to bind to diseased arterial tissue irrespective of the underlying inflammatory process. In abdominal aortic aneurysms, ^18^F-NaF PET/CT predicts increased rates of growth and important clinical end-points, such as rupture or the requirement for repair.

Much work remains to be done to bridge the gap between detecting aortic inflammation in at-risk individuals and predicting adverse clinical events. Novel radiotracers may hold the key to improve our understanding of vessel wall biology and how this relates to patients. Combined with established clinical and morphological assessment techniques, PET imaging promises to improve disease detection and clinical risk stratification.

## Introduction

Aortic diseases have varying clinical presentations and equally diverse underlying mechanisms. However, inflammation plays an important role in most major aortic pathologies, leading to degradation of the vessel wall and potentially vessel occlusion, aneurysm formation or dissection. Each represents a potentially catastrophic cause of morbidity and mortality for patients that frequently occurs without clinical warning.

The ability to monitor aortic inflammation accurately and non-invasively would therefore represent a major advance. Serum markers are non-specific to aortic inflammation and are unable to identify focal areas of disease or injury. Furthermore, whilst modern imaging techniques may detect aortic morphology and periaortic changes with great anatomical detail, they are unable to inform on the biological activity within the aortic wall itself. Metabolic imaging has the potential to meet this important unmet clinical need. Imaging probes can be used to identify inflammation directly or closely associated processes, allowing disease activity to be measured with imaging techniques such as positron emission tomography.

A combination of morphological analysis and molecular imaging presents an opportunity to detect and characterize aortic disease better. In this review, we will explore emerging molecular techniques in this field, describe the established evidence of their utility and discuss their potential to improve risk stratification and enable targeted therapy to improve patient care and outcomes.

## Pathophysiology of aortic wall inflammation

Aortic inflammation occurs in response to tissue injury and cellular stress. Repair and remodelling pathways trigger pro-inflammatory mechanisms that lead to further tissue damage. We focus on the inflammatory processes and key pathological mechanisms that affect the aorta. These include atherosclerosis, aneurysmal formation and autoimmune aortitis.

Atherosclerotic disease is an abnormal immune-mediated response to modified subendothelial lipoproteins. The result is atheroma formation within the intima of large and medium-sized arteries, including the aorta. Vulnerable plaques consist of a lipid-rich necrotic core surrounded by activated immune cells. There is a loss of extracellular matrix proteoglycans and collagen, along with the deposition of calcium. A fibrous cap attempts to contain this necrotic content and is composed of varying quantities of migratory smooth muscle cells, endothelial cells and calcification. Plaque rupture occurs when the fibrous cap fractures, instigating an acute inflammatory cascade and resulting in rapid thrombus formation. In the aorta, distal thromboembolism causes visceral or limb ischemia. Alternatively plaque rupture can be sub-clinical, resulting in plaque healing and growth. Progressive disease causes luminal stenosis, restricting flow to distal organs and potentially causing ischemic symptoms.

**Fig. 1:**
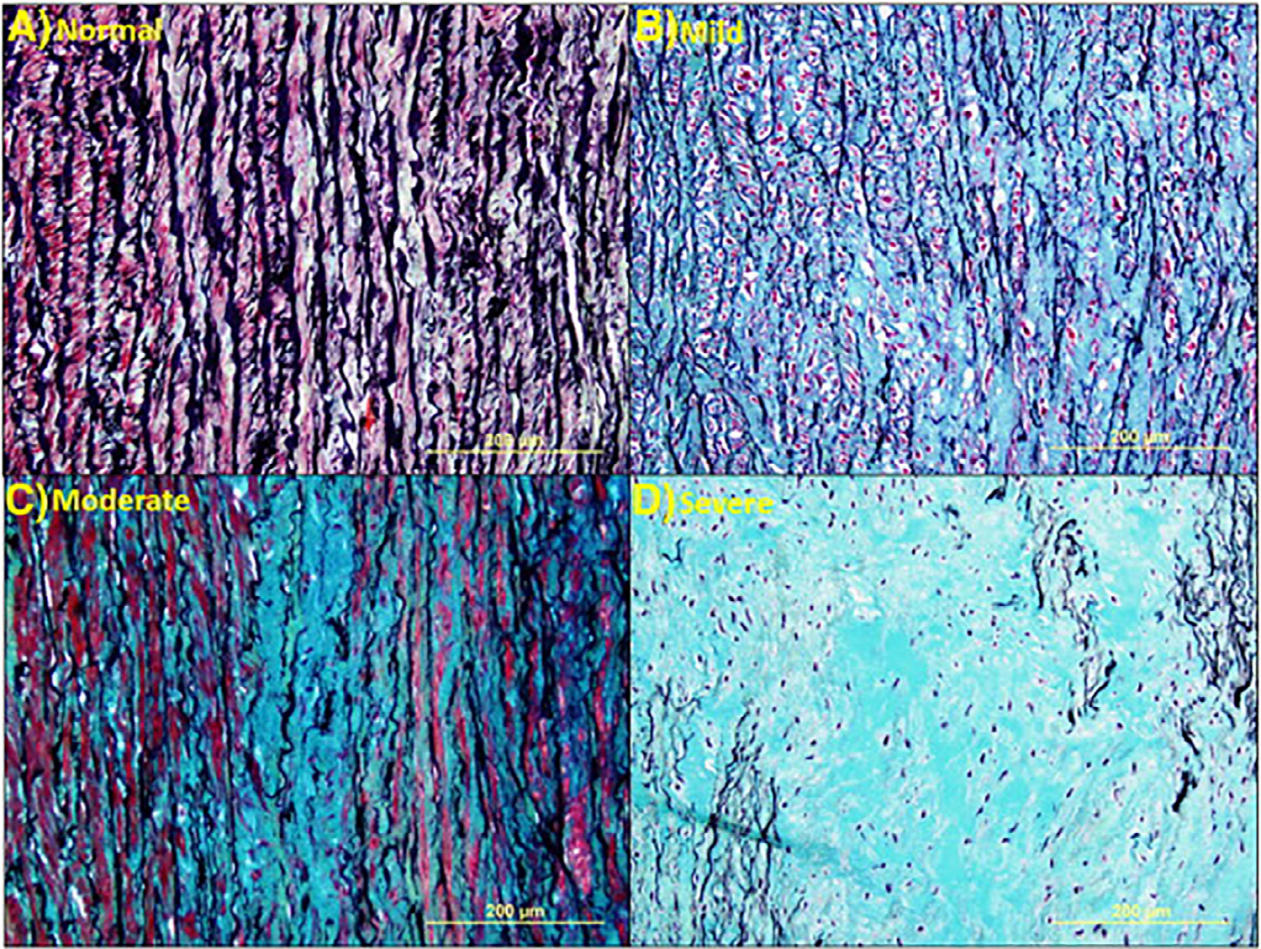
**Medial degeneration.** Histologic images showing grades of medial degeneration. (**A**) Normal aorta. (**B**) Mild medial degeneration characterized by pooling of proteoglycan between elastic lamellae. (**C**) Moderate medial degeneration with focal loss of elastic lamellae and proteoglycan deposition. (**D**) Severe medial degeneration with marked loss of elastic lamellae, SMCs, and extensive proteoglycan deposition. All Movat pentachrome stain [1].

Aortic aneurysm formation includes an inflammatory mediated process but is distinct from atherosclerosis because it is characterized by media atrophy rather than intimal proliferation (Fig. 1) [1], [2]. Consequent thinning, weakening and stiffening of the aortic wall leaves it vulnerable to dilatation and rupture.

Autoimmune aortitis, such as Takayasu and giant cell disease, is far less common than atherosclerosis and aortic aneurysm formation. Large vessel vasculitis is characterized by inflammatory infiltrates within the aortic adventitia and vasa-vasorum. The chronic inflammatory course of vasculitis leads to progressive intimal wall thickening that causes vessel stenosis or occlusion [3].

### Immune-mediated inflammation

**Fig. 2:**
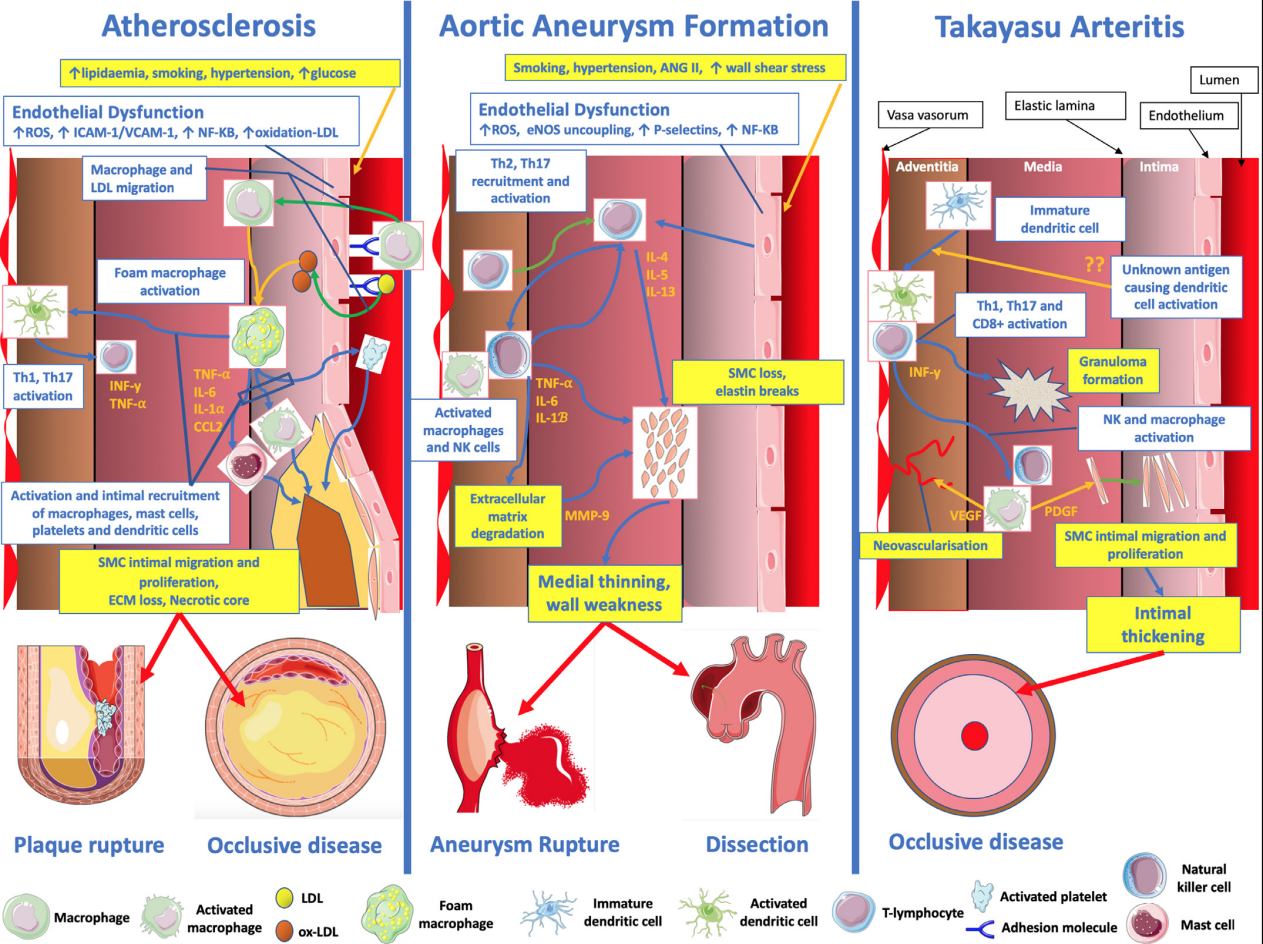
Summary of the principle underlying inflammatory mechanisms and subsequent clinical manifestations affecting atherosclerosis, aortic aneurysm formation and aortitis. eNOS = endothelial nitric oxide synthase, INF = interferon, IL = Interleukin, LDL = low density lipoprotein, MMP = Matrix metalloproteinase, NK = natural killer, NF-KB = nuclear factor kappa-light-chain-enhancer of activated B cells, ox = oxidized, ROS = reactive oxygen species, PDGF = platelet derived growth factor, SMC = smooth muscle cell, Th = T-helper, TNF = Tumour necrosis factor, VEGF = vascular endothelial growth factor. *Images adapted from*https://smart.servier.com*(Servier Medical Art by Servier) used under a creative commons license.*

In atherosclerosis, macrophage and smooth muscles cells endocytose modified-lipoproteins to become foam cells (Fig. 2). Macrophage foam cells in the intima produce pro-inflammatory cytokines such as tumor necrosis factor-γ, interleukin (IL)-1β, IL-6 and chemokine CCL2: stimuli that recruit macrophages, mast cells, platelets, T-lymphocytes and dendritic cells. All of these contribute to ongoing inflammation [4]. Local antigen presentation activates an adaptive immune response that includes cytotoxic CD8+ T-cells. These are implicated in smooth muscle and macrophage cell death [5]. T-helper cells (CD4+), specifically Th1 subcategories, contribute to overall inflammation by producing cytokines including interferon-γ and tumor necrosis factor [6].

In contrast, inflammation in aortic aneurysms primarily affects the media and adventitia. The chief immune mediator in aortic aneurysm formation appears to be CD4+ T-lymphocytes through the production of interferon-γ [7]. Whereas Th1 cells contribute to atherosclerosis, Th2 cells are strongly implicated in aneurysmal disease. These produce IL-4, IL-5 and IL-13, which stimulate natural killer cells to produce matrix metalloproteinases (MMP) and cause medial smooth muscle atrophy [2]. Th17 are common to both, atherosclerotic and aneurysmal disease. They promote macrophage activation by releasing tumor necrosis factor-α, IL-6 and IL-1. These cytokines have downstream effects that promote MMP-9 expression and smooth muscle loss [8].

Similar to aortic aneurysm formation, Takayasu aortitis is an adaptive immune response with infiltration including Th1 and Th17 cell subtypes. The initial trigger is unknown, although mycobacterial-related heat shock protein is speculated [9]. Unlike aortic aneurysm formation, inflammatory lesions in Takayasu aortitis are largely formed in the adventitia via the vasa-vasorum [9]. Medial inflammation driven by macrophages expressing vascular endothelial growth factor and platelet-derived growth factor contribute to neovascularization and smooth muscle proliferation and migration to the intima – the primary cause of severe intimal thickening [9].

### Vascular calcification

Vascular calcification is a healing response to inflammation and tissue degradation. Detection of the early stages of microcalcification therefore acts as a surrogate of inflamed damaged aortas. By comparison, detection of the latter stage of macrocalcification is associated with inflammation that is healing or burnt out.

Vascular calcification is characterized by the initial deposition of microscopic calcium- and phosphate-containing hydroxyapatite crystals. Macrophages produce calcifying matrix vesicles that contribute to intimal microcalcification in atherosclerosis [10]. Smooth muscle cells that migrate to the intima also undergo osteoblastic change and encourage intimal microcalcification [11]. Deposits of microcalcification within the media are associated with elastin breaks in both chronic kidney disease and aneurysmal aortic specimens in patients with connective tissue disorders [12].

## Detecting aortic inflammation

Aortic inflammation is progressive and silent. Symptoms do not occur until advanced stages of disease and often signal impending catastrophe. Hence, the early detection of aortic inflammation would be pivotal to identify and monitor disease as well as direct early therapy. In aortitis, biological drugs used to target aortic inflammation are potentially toxic. Newer anti-inflammatory drugs are effective but are expensive and there is a need to ensure treatment is directed to the right patient.

### Assessing aortic diameter and wall thickness

Aortic remodelling alters the shape of the aorta, which is best detected with ultrasound, computed tomography (CT) or magnetic resonance imaging (MRI). These modalities are the cornerstone of modern aortic imaging in contemporary clinical practice (Table 1).

**Table 1:** The strengths of different aortic imaging modalities.

	Imaging modality			
	Duplex ultrasound	Computed tomography	Magnetic resonance imaging	Positron emission tomography
Aortic morphology	+	++	+	
Luminal morphology	+	++	++	
Aortic wall thickness	+	+	++	
Metrics of blood flow	++		+	
Calcification		++		
Thrombus characterisation			++	Novel thrombus and fibrin targeting radiotracers
Glucose metabolism				++
				^18^F-fluorodeoxy-glucose
Macrophage infiltration			++	++
			USPIO	^18^F-DOTATATE, VCAM-1, ^11^C-Choline,^11^C-PK11195
Microcalcification				++
				^18^F-Sodium Fluoride
Vessel wall angiogenesis	+			+
	Contrast-Enhanced US			^18^F-Galacto-RGD

[++] – Ideally suited; [+] – Detection is possible.

Ultrasound is the screening modality of choice for sub-diaphragmatic aortic disease owing to its reproducible results, wide availability and relative affordability. Duplex ultrasonography can detect the morphology of the abdominal aorta and its branches, along with changes in the velocity of blood flow. Since ultrasound cannot penetrate bony structures, visualizing the thoracic aorta requires a transesophageal probe. However, more detailed techniques are required to distinguish morphological changes specific to inflammation.

Catheter angiography outlines the aortic lumen with exceptional detail. However, it is invasive and associated with potential complications including arterial injury, radiation exposure and nephrotoxicity. Non-invasive imaging techniques are a preferred alternative to catheter angiography because they image the whole aorta with minimal risk of iatrogenic complications. Modern CT and MRI have excellent spatial resolution and can accurately detect luminal irregularities. They also obtain images of the vessel wall and associated peri-aortic tissue. MRI has better soft tissue characterization than CT and is better at assessing wall thickness [13].

CT is well suited to detect established calcified plaque owing to its reliance on x-rays for image acquisition. Modern CT scanners can detect mature plaques and CT texture analysis offers an objective approach to analyzing individual lesions. The total burden of vessel calcification can be quantified with a calcium score. Calcium scores are a condensed, yet powerful, metric that provide objectivity to the heterogenous patterns of calcification within the aorta. These scores can be used in risk stratification models to predict disease progression.

Both, CT and MRI, reliably detect aortic changes when the wall is thickened as is seen in vasculitis [14]. The inflamed artery appears as a ring of high attenuation on arterial phase CT imaging within a thickened low-attenuation aortic wall (Fig. 4) [15]. The inflamed wall is frequently littered with spotty calcification and the calibre of the vessel itself may be increased. These high-risk features signal active disease that is congruent over large sections of the aorta.

### Serum markers of disease activity

Inflammatory mediators produced in diseased aortic wall, such as interleukins and matrix metalloproteinases, are elevated in the serum of patients with aortopathy [16]. These likely represent global vascular inflammation and are better suited to assessing aortitis compared to atherosclerotic or aneurysmal disease [17].

Immunoglobulin-G subtype 4 (IgG-4) is increasingly being recognized as a potent mediator of autoimmune aortitis. Serum IgG-4 upregulates pro-inflammatory interleukins and is markedly increased in “inflammatory” aneurysms [16]. High IgG-4 concentrations are associated with larger aortic sizes [18] and up to a quarter of patients with IgG-4 related disease progress to develop aneurysms [19]. Serum c-reactive protein and erythrocyte sedimentation rates in these patients indicate the burden of global vascular inflammation and allows the monitoring of therapy.

### Imaging biological activity within the aortic wall

Non-invasive imaging allows inflammatory activity to be measured specifically within the aorta, thereby overcoming many of the limitations associated with serum biomarkers. Novel biological radiotracers detect specific disease processes and the pattern of radioactive decay can be detected using positron emission tomography (PET), which offers sensitive detection of radiotracers and produces a topological map of tracer binding. PET is inherently low-resolution. For this reason, it is combined with CT or MRI for anatomical context.

Metal-based “smart contrast agents” used with magnetic resonance imaging can identify inflammatory changes within vascular tissue. Targeting vascular biological activity in this way offers more complete insight to aortic wall disease compared to conventional anatomical-based imaging used routinely in clinical practice.

#### MRI sensitive smart contrast agents

**Fig. 3:**
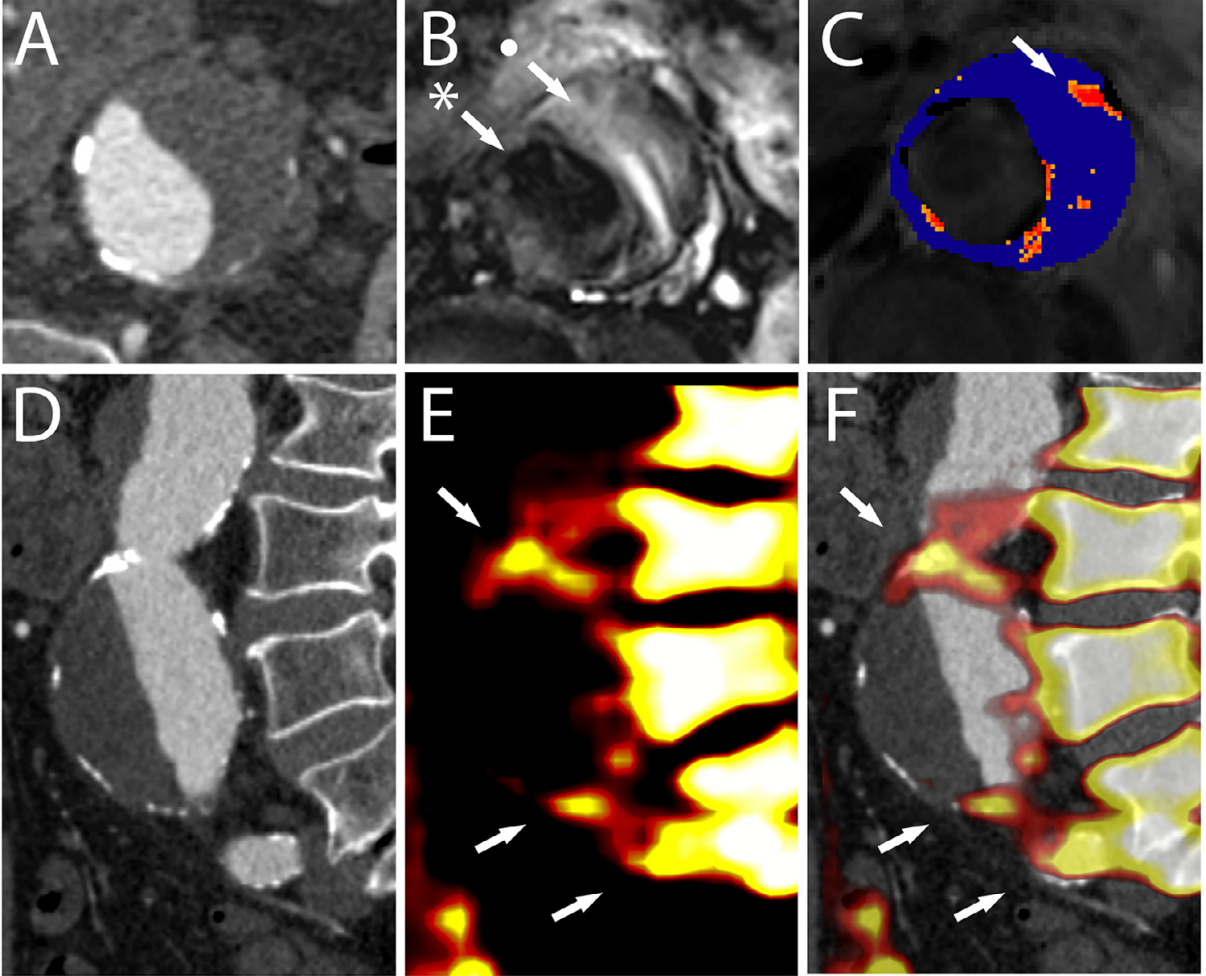
**Computed tomography, magnetic resonance imaging and positron emission tomography in a patient with an abdominal aortic aneurysm**. Axial view of the aneurysm as seen on computed tomography (**A**) shows a sac with thrombus and calcified plaque in the aortic wall. T2-weighted magnetic resonance imaging (**B**) can differentiate between the lumen (*), thrombus (•) and adjacent structures. T2* magnetic resonance imaging (**C**) shows high ultrasmall particles of iron oxide uptake (arrow) in the wall of the aneurysm. The sagittal computed tomography view (**D**) delineates the morphology of the aneurysm. 18F-Sodium Fluoride uptake (**E**) seen anterior to the vertebral body (arrows). Superimposing positron emission tomography signals over the computed tomography images (**F**) confirms high 18F-Sodium Fluoride uptake at the aneurysm neck, bifurcation and left common iliac artery (arrows). (For interpretation of the references to colour in the text, the reader is referred to the web version of this article.)

One approach to visualize aortic inflammation is to image cellular activity within the aortic wall directly. Ultrasmall superparamagnetic particles of iron oxide (USPIO) are sub-nanometre compounds that are engulfed by tissue-resident macrophages. They have been used to demonstrate macrophage infiltration within the aortic wall owing to their ability to cause a rapid decline in T2*. This signal change can be represented on visual colour maps by obtaining pre- and post-USPIO MRI sequences (Fig. 3B–C) [20]. The degree of signal decay quantifies USPIO accumulation and is a surrogate marker of macrophage activity.

**Fig. 4:**
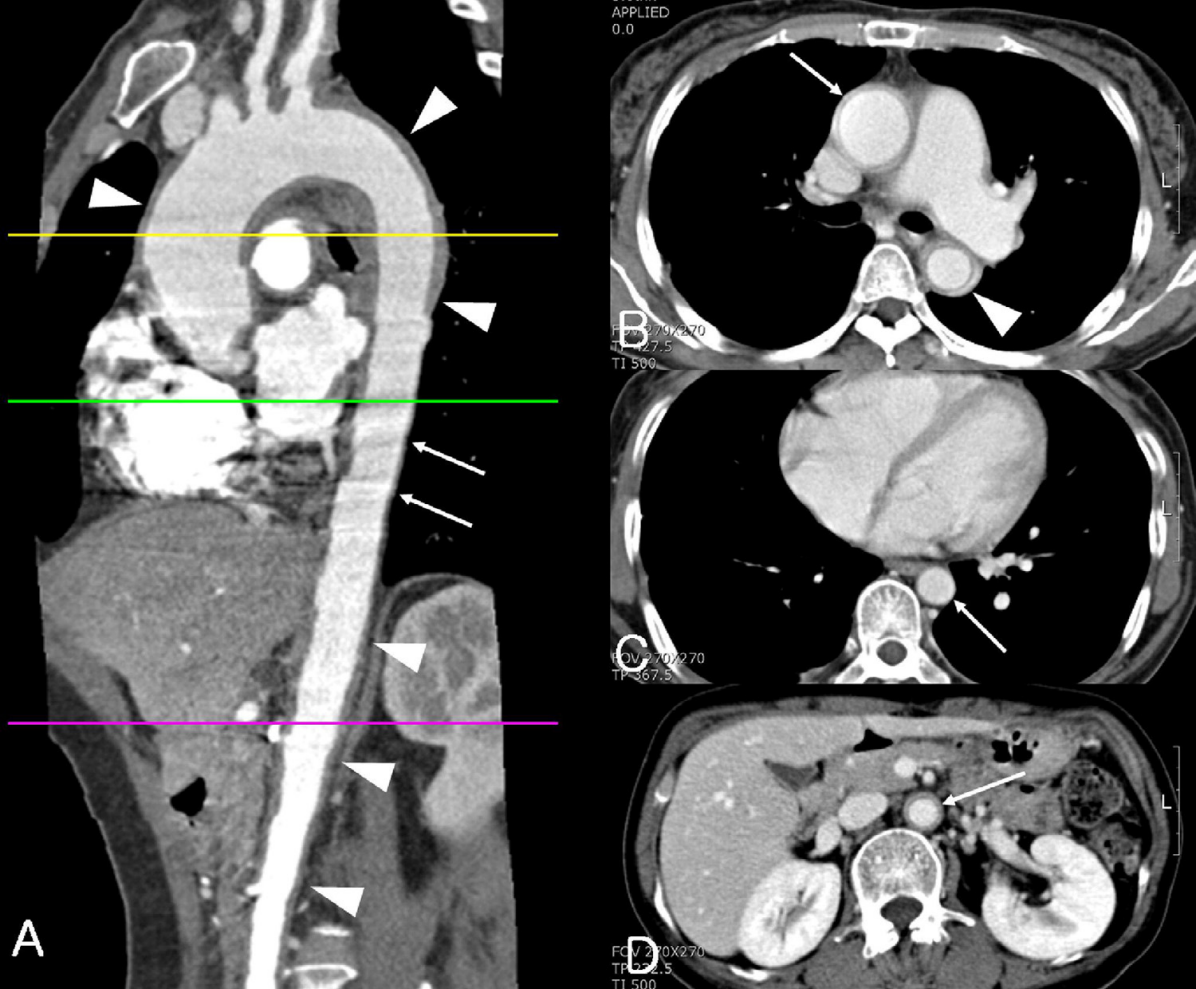
**Takayasu arteritis involving aorta with skipped segment in a 50-year-old woman.** (**A**) Multiplanar reformation image shows wall thickening (arrowheads) of ascending thoracic aorta, aortic arch, proximal descending thoracic aorta, and abdominal aorta. No wall thickening of distal descending thoracic aorta is noted. Motion artifact (pulsation artifact) was not observed in the thickened segment such as aortic arch and abdominal aorta due to stiffness of involved aorta and was observed in the distal descending thoracic aorta (arrows) due to pulsation of the noninvolved segment. (**B**) CT scan at the proximal descending thoracic aorta shows diffuse wall thickening and inner low attenuated ring of ascending (arrow) and descending thoracic aorta (arrowhead). (**C**) CT scan at the distal descending thoracic aorta (arrow) shows no wall thickening of aorta. (**D**) CT scan at the abdominal aorta shows diffuse wall thickening and inner low attenuated ring of abdominal aorta (arrow) [15].

Tropoelastin is a soluble precursor that cross-links elastin. Its presence signifies elastin regeneration following extracellular matrix injury. Tropoelastin is not detectable in healthy tissue. Gadolinium-based contrast media can target tropoelastin proteins and have been validated in animal aortic models and injured myocardium [21], [22]. The presence of these proteins is associated with focal weakening of the arterial wall and correlates with rupture sites in mouse models of aneurysms.

#### ^18^F-Fluorodeoxyglucose

^18^F-Fluorodeoxyglucose (^18^F-FDG) is a glucose analogue that is trapped in cells with increased glycolytic activity, such as inflamed aortae. Because ^18^F-FDG reflects global metabolic activity, its binding is non-specific and overlaps between diseased and healthy arterial tissues. In low-grade inflammatory conditions, such as atherosclerosis and aneurysm formation, ^18^F-FDG PET is less able to detect active disease reliably.

On the other hand, aortitis reflects a much more aggressive form of vascular inflammation affecting both the intima and media. Marked vessel wall hypertrophy causes arterial stenosis and can progress to complete occlusion. Morphological imaging is the basis of diagnosing vascular complications in aortitis and CT is highly sensitive in detecting diseased vessels in patients with large vessel vasculitis [23]. However, per-segment analysis of large vessels misses up to 40% of individual lesions [24]. ^18^F-FDG PET/CT improves the detection of aortitis beyond CT alone by detecting inflamed sections that look normal on CT. ^18^F-FDG PET is also influenced by glucocorticoid and immunosuppressant therapy, which can be used to monitor the efficacy of treatment. However, this is a double-edged sword because late ^18^F-FDG PET scans may miss vessel involvement if therapy has started [25].

The true sensitivity of ^18^F-FDG PET in vasculitis is unknown. Longitudinal studies report that nearly half of vasculitis patients with biopsy proven disease do not exhibit significant ^18^F-FDG uptake and this proportion reduces further with time [26]. Although ^18^F-FDG PET/CT may help detect inflammatory aortic disease, it is not robust enough to be used in isolation. Indeed, biopsy remains the gold standard for diagnosing large vessel vasculitis.

#### Novel inflammatory PET radiotracers

A number of radiotracers have been developed to detect specific biological processes. Direct leukocyte detection is possible by targeting CXCR4 receptors expressed on the surface of multiple types of inflammatory cells. Aneurysmal aortic tissue has upregulated CXCR4 receptors within its aortic wall and animal studies show that ^68^Ga-Pentixafor binds to CXCR4 receptors which localize to areas of increased aortic wall disease [27].

Whereas CXCR4 is expressed by a range of pro-inflammatory leukocytes, it is also possible to target specific cell lines. For instance, the translocator proteins (TSPO) are 18-kDa glycoproteins expressed on the mitochondria of activated macrophages in much larger quantities than other cells [28]. The TSPO ligands can be radiolabelled and have been tested in human atherosclerotic tissue, where they accurately identify culprit lesions and macrophage infiltration. Aortic studies also show marked uptake of TSPO radiotracers in vasculitis [28]. However, these findings have not translated to detect low-grade inflammation in aortic aneurysms [29].

Somtatostatin receptor subtype 2 (SST2) receptors are highly specific to activated macrophages. Variations in SST2 analogues combine a DOTA- or NOTA- cage with a ^68^Ga or ^64^Cu radioisotope attached to a -TATE or -NOC somtatostatin ligand. ^68^Ga-DOTATATE ([^68^Gallium-DOTA0-Tyr^3^]octreotate) is the most studied of these and binds preferentially to atherosclerotic plaques with high macrophage infiltration [30].

Degraded aortic tissue exhibits abnormal angiogenesis and is present in abundance at sites of aortic rupture [31]. ^18^F-Galacto-RGD binds to α_v_β_3_ receptors that stimulate angiogenesis, typically in hypoxic environments [32]. This experimental agent has been validated in carotid and coronary atherosclerotic plaque, where it adheres strongly to lesions with a rich network of vasa vasorum.

### ^18^F-Sodium Fluoride

Detecting microcalcification requires a different approach from established calcified plaque because CT does not have sufficient resolution to visualise the tiny calcium-containing crystals deposited within the arterial wall. ^18^F-Sodium fluoride (^18^F-fluoride or ^18^F-NaF) is a promising radiotracer that binds to hydroxyapatite crystals deposited during microcalcification [11].

In the vascular system, ^18^F-NaF binds to microcalcification with great affinity [33]. This is true in nearly all metabolic processes that lead to vessel calcification, including atherosclerosis and medial degeneration [33]. The Sodium Fluoride in Abdominal Aortic Aneurysm (SoFIA^3^) study explored the role of ^18^F-NaF in 72 patients with abdominal aortic aneurysms [34]. It showed that aneurysmal aorta exhibit markedly increased ^18^F-NaF uptake compared to non-diseased segments. Comparisons of ^18^F-NaF binding between dilated and normal calibre aortae revealed increased tracer uptake in aneurysmal segments (log2 radiotracer uptake 1.647 ± 0.537 vs. 0.881 ± 0.414; difference 0.766; 95% CI: 0.517 to 1.011; *p* < 0.0001) (Fig. 3E–F). This binding pattern was independent of the aortic calcium score and aortic diameter. Histological comparisons of aortic tissue obtained following open aneurysm repair showed that ^18^F-NaF binding correlates with microcalcification and aortic degradation in these subjects.

Vessel wall calcification is universal in many aortopathies including aortic dissections and connective tissue disorders. The role of ^18^F-NaF PET in these diseased states remains of great interest in vascular imaging.

## Risk stratification

Studying the biology within aortic tissue offers a new perspective on aortic diseases. Morphological change and established calcified plaque are late manifestations of aortic wall disease. A combination of anatomical imaging with the pathological processes seen using PET offers a more complete assessment of diseased aortic state. Together, these modalities promise to improve risk prediction models.

### Aortic expansion

Aortic expansion is sporadic and is accelerated at larger diameters. The aortic wall continues to weaken as the aorta enlarges, potentially leading to rupture. However, aortic size alone is unreliable because sub-threshold aneurysms may also rupture. Alternatively, others remain intact despite significant growth. Clinical practice is guided by historical data that relies on late features such as diameter change as measured on ultrasound or cross-sectional anatomical imaging [35].

**Fig. 5:**
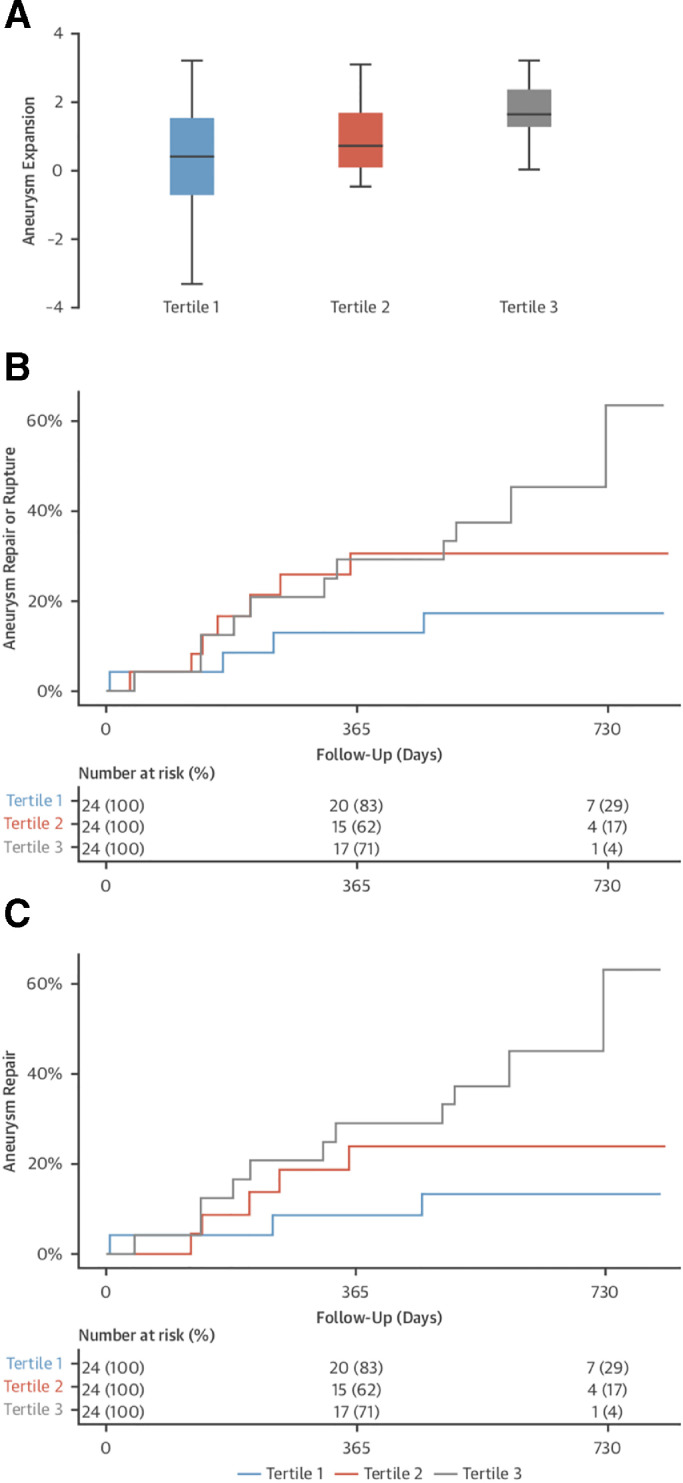
**Association of 18F–sodium fluoride (18F–NaF) uptake with disease progression and clinical outcome.** (**A**) Rate of aneurysm expansion (millimeters per year, log2 transformed) across the tertiles of 18F-NaF uptake. The highest tertile expanded more rapidly than those in the lowest tertile (3.10 vs. 1.24 mm/year, respectively, *p* = 0.008). Cumulative event rate (censored at date of death) across the tertiles of 18F–NaF uptake for (**B**) abdominal aortic aneurysm repair or rupture (log-rank *p* = 0.043) and (**C**) abdominal aortic aneurysm repair (log-rank *p* = 0.014). *Adapted from Forsythe RO, Dweck MR, McBride OMB, Vesey AT, Semple SI, Shah ASV,* et al. *18F–Sodium Fluoride Uptake in Abdominal Aortic Aneurysms: The SoFIA3 Study. Journal of the American College of Cardiology. 2018 Feb 6;71(5):513–23.*

The approach of managing aortic aneurysmal disease using a blanket threshold for intervention is inadequate. For instance, it does not account for variations in normal aortic diameter due to sex and ethnicity. Biological imaging provides insight to the pathological mechanisms driving aortic expansion. In the SoFIA^3^ study [36], individuals that had the highest uptake of ^18^F-NaF in the most-diseased aneurysmal segment also had the fastest rate of aortic growth (log_2_^18^F-NaF uptake *r* = 0.365; *p* = 0.006) and composite end-point of rupture or repair (log-rank *p* = 0.043) (Fig. 5) [37]. This finding remained true independent of potential confounding factors such as aortic size and clinical risk factors. The SoFIA_3_ study showed for the first time that detecting markers of aortic degeneration in aortopathy can predict aortic expansion at a much earlier timepoint than is currently possible.

Similarly, resident macrophages in the aortic wall of abdominal aortic aneurysms can be detected using USPIOs on T2*-weighted MRI. The Magnetic resonance imaging for Abdominal Aortic Aneurysms to predict Rupture or Surgery (MA^3^RS) study was the first to prospectively study resident aortic macrophages in a large clinical cohort (*n* = 342). There were 140 clinical events consisting of 126 aneurysm repairs and 17 aortic ruptures. Survival analysis showed increased aneurysm repair or rupture (log rank *p* = 0.028) and a trend towards aneurysm-related mortality (log rank *p* = 0.059) when USPIO uptake was high [20].

Weakening of the aortic wall is a feature of other aortopathies too, such as aortic dissections or aortic manifestations of connective tissue disorders. Aortic expansion is much faster in these conditions. Again, the only clinically useful predictor of growth is aortic size. Since the thoracic aorta is most commonly affected, surveillance requires regular CT or MR imaging to predict aortic rupture. This perhaps explains why the 3-year mortality following aortic dissection approaches 1-in-4 [38]. Mortality increases rapidly in connective tissue disorders such as Marfan syndrome. Here, cardiovascular complications are responsible for up to 90% of deaths. There is a pressing need to improve our predictions of aortic expansion and rupture.

**Fig. 6:**
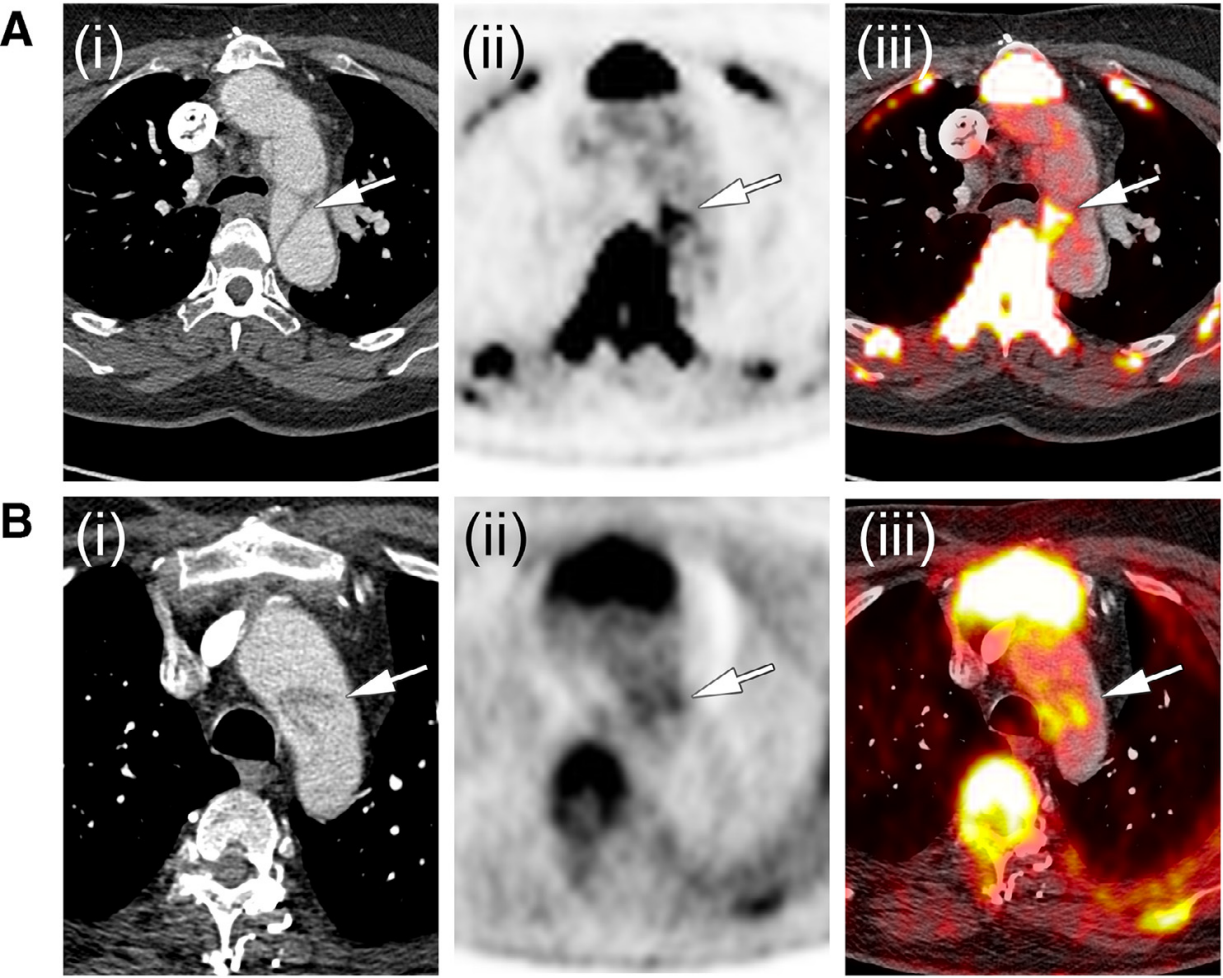
**^18^F-Sodium Fluoride following aortic dissection**. (**i**) Contrast enhanced computed tomography, (**ii**) ^18^F-Sodium Fluoride positron emission tomography and (**iii**) combined ^18^F-Sodium Fluoride positron emission tomography/ computed tomography in a patient with (**A**) hypertension-induced aortic dissection and (**B**) in a patient with Marfan Syndrome.

Preliminary data suggest focal uptake of ^18^F-NaF within the aortic wall following dissections in both, Marfan- and non-syndromic aortae (Fig. 6). Indeed, the pathological processes underlying aortic expansion in aortopathies share common pathways involving medial degeneration and calcium mineralization. Similar to abdominal aortic aneurysms, ^18^F-NaF PET/CT offers the potential to predict aortic expansion in these aortopathies as well.

### Occlusive aortic disease

Atheroma is the most common cause of chronic aortic occlusive disease. Intervention is driven by clinical symptoms such as buttock or lower limb claudication ultimately leading to rest pain and tissue loss. The strongest predictors of disease progression are clinical risk factors and management is focused on addressing these. The progression of occlusive aortic disease is highly variable and predicting disease evolution is challenging.

^18^F-NaF PET detects active plaque biology and better differentiates culprit from non-culprit lesions with greater sensitivity than ^18^F-FDG PET in the coronary and carotid vessels [39]. Plaques with high ^18^F-NaF binding exhibit high-risk features, such as dense macrophage infiltration, larger necrotic cores, thin fibrous caps and vessel remodelling [40]. Aortic atheroma shares common features with plaques found in smaller calibre vessels. ^18^F-NaF uptake in the large peripheral vessels is a marker of global calcium burden and correlates strongly with established cardiovascular risk factors [41]. The role of aortic ^18^F-NaF PET/CT in atheromatous aortic and peripheral vascular disease remains to be evaluated.

### Future direction

Current guidelines recommend regular surveillance of aortae at risk of dilatation. Ultrasound is sufficient to measure the diameter of the abdominal aorta but serial CT or MRI is required to image the thoracic aorta. Evidence to recommend routine molecular or metabolic imaging in aneurysmal aortic disease is lacking although ^18^F-FDG PET is increasingly being used for aortitis. Larger clinical studies are required to examine the true predictive value of metabolic imaging in aortopathy to predict disease progression. These investigations are resource intensive and biologically compatible radiotracers need to be manufactured in a timely fashion to prevent premature decay. For many radiotracers, this remains a significant barrier to translating to clinical practice.

^18^F-NaF PET/CT is more readily manufactured than other tracers, and has shown promising initial data to detect vessel degeneration and to predict disease progression. Collaborative efforts to facilitate larger studies on ^18^F-NaF PET are required to validate its use further in aneurysmal disease, as this is perhaps the most promising approach to date that could be readily translated into the clinic.

## Conclusion

Atherosclerosis, aneurysm formation and aortitis represent independent aortopathies that are all affected by aortic wall inflammation. Atherosclerosis involves intimal injury and remodelling, whereas aneurysm formation results from medial degeneration. Autoimmune aortitis is characterized by both, medial infiltration and marked intimal hypertrophy. The cellular and molecular processes leading to these aortic diseases are varied but also share common pathways such as vessel wall calcification. Here, microcalcification is an early pathological feature that occurs in response to cellular destruction and signifies intense active disease.

Detecting early microcalcification using ^18^F-NaF PET/CT holds great promise because it identifies necrotic material within vascular beds that drives further inflammation. In abdominal aortic aneurysms, ^18^F-NaF PET/CT identifies aneurysms that grow rapidly or require surgical repair. Similarly, in atherosclerotic disease, ^18^F-NaF identifies culprit plaques following cap rupture.

Metabolic imaging and the emergence of novel radiotracers, such as SST2 and TSPO ligands, means that we can non-invasively identify metabolic activity related to inflammatory processes before morphological changes manifest. The combination of metabolic imaging with established anatomical imaging offers a more complete assessment of inflammation, improves risk stratification and may ultimately guide therapy.
